# Voltammetric Determination of Codeine on Glassy Carbon Electrode Modified with Nafion/MWCNTs

**DOI:** 10.1155/2015/626458

**Published:** 2015-02-09

**Authors:** Robert Piech, Martyna Rumin, Beata Paczosa-Bator

**Affiliations:** Faculty of Material Science and Ceramics, AGH University of Science and Technology, Mickiewicza 30, 30059 Cracow, Poland

## Abstract

A glassy carbon electrode modified with a Nafion/MWCNTs composite is shown to enable the determination of codeine using differential pulse voltammetry in phosphate buffer of pH 3.0. At a preconcentration time of 15 s, the calibration graph is linear in the 0.5 *µ*M (0.15 mg·L^−1^) to 15 *µ*M (4.5 mg·L^−1^) concentration range with a correlation coefficient of 0.998. The detection limit at a preconcentration time of 120 s is as low as 4.5 *μ*g·L^−1^. The repeatability of the method at a 0.6 *μ*g·L^−1^ concentration level, expressed as the RSD, is 3.7% (for *n* = 5). The method was successfully applied and validated by analyzing codeine in drug, human plasma, and urine samples.

## 1. Introduction

Codeine is a naturally occurring opium alkaloid. It is chemically known as 7,8-didehydro-4,5-epoxy-3-methoxy-17-methylmorphinan-6-ol monohydrate [1] but is less potent than morphine, with a potency ratio of 1 : 10 [2]. Codeine is considered as a prodrug, metabolized to active compounds of morphine and codeine-6-glucuronide [3]. It is traditional choice for the treatment of mild and moderate pain [4] and is frequently recommended for pediatric use [5]. Moreover, codeine is widely used in cough cold syrup, but it would cause drug addiction and make mental damage to patient if abused [6]. Thus a sensitive, specific, fast, and cheap method of determining codeine is necessary for studying the presence of codeine in drugs and human body fluids.

The analytical methods most often used to determine codeine in various samples are high performance liquid chromatography [7–9], spectrophotometry [10, 11], gas chromatography [12], and capillary electrophoresis [13]. However, these methods are usually time consuming. Electrochemical methods for the determination of codeine as potentiometry and polarography are also reported [14, 15] but these methods are of rather low sensitivity. In the group of more sensitive methods as voltammetry various working electrodes such as glassy carbon electrode (the detection limit not shown) [16], Nafion/ruthenium oxide pyrochlore CME chemically modified electrode with the detection limit of 10 nM [17], clay-modified screen-printed carbon electrode (CMSPE) with the detection limit of 20 nM [18], Prussian blue film modified-palladized aluminum electrode (PB/Pd/Al) with the detection limit of 0.8 *μ*M [19], aluminum electrode modified with a thin layer of metallic palladium (Pd/Al) with the detection limit of 5 *μ*M [20, 21], boron-doped diamond film electrode with the detection limit of 80 nM [22], and single-walled carbon nanotubes modified carbon ceramic electrode (SWCNTs/CCE) with the detection limit of 110 nM [23] are used.

The aim of this work was to study the high sensitive determination of codeine by means of linear sweep voltammetry (LSV) and differential pulse voltammetry (DPV) with the use of glassy carbon (GC) electrode modified with Nafion/multiwalled carbon nanotubes (MWCNTs). The new procedure was examined and successfully used for the determination of a low codeine concentration in urine, human blood plasma, and medical products. Potential interference from selected metal ions, ascorbic acid, citric acid, and surface-active substances was checked.

## 2. Material and Methods

### 2.1. Measuring Apparatus and Software

A multipurpose Electrochemical Analyzer M161 with the electrode stand M164 (MTM-ANKO, Poland) was used for all voltammetric measurements. The classical three-electrode quartz cell, volume 20 mL, consists of a GC electrode (diameter 3 mm, Mineral, Poland) modified with Nafion/MWCNTs as the working electrode, a double junction reference electrode Ag/AgCl/KCl (3 M) with replaceable outer junction (3 M KCl), and a platinum wire as an auxiliary electrode. pH measurements were performed with a laboratory pH-meter (N-512 elpo, Polymetron, Poland). Stirring was performed using a magnetic bar rotating at approximately 500 rpm. All experiments were carried out at room temperature. The MTM-ANKO* EAGRAPH* software enabled electrochemical measurements, data acquisition, and advanced processing of the results.

### 2.2. Chemicals and Glassware

All reagents used were of analytical grade. KH_2_PO_4_ and K_2_HPO_4_ were obtained from Merck and H_3_PO_4_ was obtained from CHEMAN (Poland). In measurements a 0.1 M phosphate buffer solution (pH 3.0) was used (prepared using quadruply distilled water). Standard stock solutions of codeine (0.01 M) were prepared by dissolving codeine phosphate (local source) in distilled water. Solutions with lower codeine concentrations were made by appropriate dilution of the stock solution. The multiwalled carbon nanotubes (purity > 95%, diameter 40–60 nm, and length 5–15 *μ*m) were obtained from Nanostructured & Amorphous Materials Inc. (USA). Nafion 5 wt.% solution in a mixture of lower aliphatic alcohols and water was purchased from Aldrich.

Prior to use, glassware was cleaned by immersion in a 1 : 1 aqueous solution of HNO_3_, followed by copious rinsing in distilled water.

### 2.3. Preparation of the Electrode

Prior to modifying the GC electrode was mechanically polished with Al_2_O_3_ (0.05 *μ*m) and then rinsed and sonicated 5 min in distilled water. Next 10 mg of MWCNTs was added to 10 mL ethanol and Nafion (final Nafion concentration 0.1%) and then sonicated for 2 hours to obtain a homogenous suspension. The prepared GC electrode was coated with 10 *μ*L homogenous Nafion/MWCNTs and allowed evaporating the solvent at room temperature in the air. Nafion/MWCNTs GC electrode was conditioned in phosphate buffer (pH 3.0) and can be used for several measurements.

### 2.4. Standard Procedure of Measurements

The electrochemical behavior of the Nafion/MWCNTs glassy carbon modified electrode was investigated using cyclic voltammetry. The voltammograms were recorded in the potential range from 875 to 1425 mV. Before each registration scan the potential of 1450 mV (2 s) was applied to clean the surface of the electrode. The electrode conditioned in this way was used to determine codeine in the supporting electrolyte: 0.1 M phosphate buffer (pH 3.0) (total volume 10 mL) contained in a quartz voltammetric cell. In the case of DP measurements the potential of the electrode was changed in the following sequence: cleaning potential 1350 mV for 2 s and preconcentration potential *E*_acc_ = 300 mV for *t*_acc_ = 20 s. During the preconcentration step codeine was adsorbed while the solution was being stirred (ca. 500 rpm) using a magnetic stirring bar. Then, after a rest period of 5 s a differential pulse voltammogram was recorded in the anodic direction from 300 to 1350 mV. The other experimental parameters were as follows: step potential, 5 mV; pulse potential, 50 mV; time step potential, 40 ms (20 ms waiting + 20 ms sampling time). Measurements were performed in solutions not previously treated with deoxygenation. Quantitative measurements were performed using the standard addition procedure.

### 2.5. Sample Preparation

#### 2.5.1. Urine and Human Blood Plasma

For DPV determination of codeine in urine, 250 *μ*L of the fresh sample was added directly into voltammetric cell with supporting electrolyte (total volume 10 mL). In the case of human blood plasma, 50 *μ*L of the fresh plasma was added to the supporting electrolyte (total volume 10 mL). The samples (originally drug-free) were spiked with codeine.

#### 2.5.2. Tablet and Syrup

For the determination of codeine in tablet, 3 tablets (15 mg codeine per tablet) were dissolved in 25 mL volumetric flask and additionally sonicated for 15 min. Next 10 *μ*L of the sample was added to the voltammetric cell.

For the determination of codeine in syrup (15 mg codeine per 10 mL syrup), 10 *μ*L of the syrup was added directly to the voltammetric cell.

## 3. Results and Discussion

### 3.1. Cyclic Voltammetry Studies

**Figure 1:**
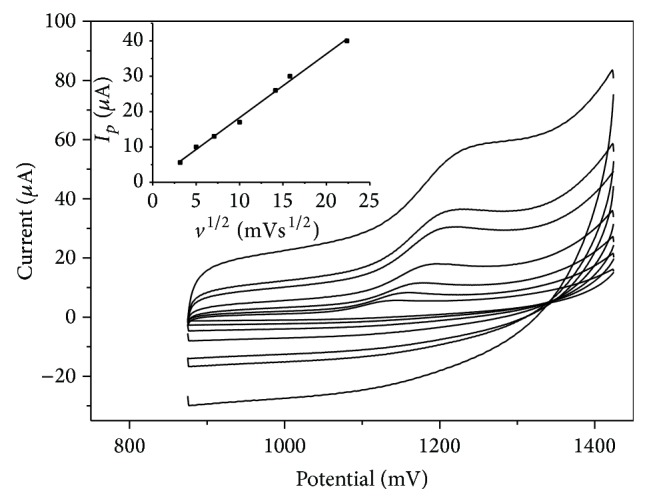
The cyclic voltammograms obtained for 50 *μ*M codeine at the GC electrode modified with 10 *μ*L Nafion/MWCNTs in 0.1 M phosphate buffer (pH 3.0). Scan rate in the range from 10 to 500 mV s^−1^ (inner figure: dependence of the codeine peak current on square root of scan rate).

The influence of the scan rate (*v*) on the peak current and peak potential at the GC electrode modified with Nafion/MWCNTs was investigated in the range of 10 mV s^−1^ to 500 mV s^−1^ (Figure 1). During the scan at pH of 3.0 in phosphate buffer the single anodic peak has appeared. The absence of reduction peak in the reverse step indicates the irreversibility of the electrode reaction.

The peak current versus square root of scan rate gave a straight line practically up to 500 mV s^−1^. The obtained linear regression equation is

(1) $$\begin{array}{c} I_{p}=1.80v^{1/2}+0.26\left[\mu \text{A}\right],\;\;r=0.997. \end{array}$$

This suggests that the process of electrode reaction is controlled by diffusion of codeine.

**Figure 2:**
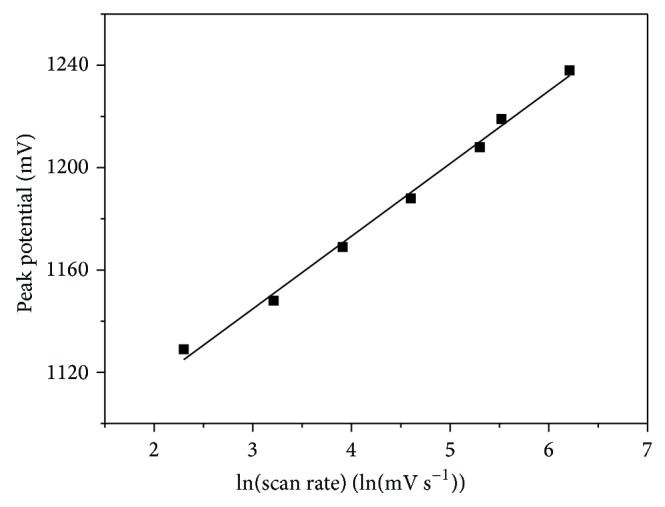
Dependence of the codeine peak potential on ln⁡⁡ of scan rate in the range from 10 to 500 mV s^−1^ for 50 *μ*M codeine in 0.1 M phosphate buffer (pH 3.0).

The anodic peak potential was shifted in the positive direction with the increasing scan rate. The peak potential versus ln⁡⁡ scan rate gave a straight line (Figure 2).

The obtained linear regression equation is

(2) $$\begin{array}{c} E_{p}=28.4\ln\left(v\right)+1060\left[\text{mV}\right],\;\;r=0.997. \end{array}$$

Based on the theory for an irreversible electrode reaction [24] from the slope of *E*_*p*_ versus ln⁡(*v*), *αn* = 0.45 could be obtained and the number of the electron transfers for *α* assuming 0.5 could be calculated to be 1.

### 3.2. Influence of DPV Parameters Technique on Codeine Peak

The important parameters of the DPV technique are pulse amplitude (Δ*E*), potential step amplitude (*E*_*s*_), waiting time (*t*_*w*_), and sampling time (*t*_*s*_). Consequently, these parameters were investigated. To optimize the conditions for codeine measurements, the following instrumental parameters were systematically varied: Δ*E* in the range 5–100 mV (both positive and negative modes), *E*_*s*_ in the range 1–7 mV, and *t*_*w*_ and *t*_*p*_ from 10 to 60 ms.

**Figure 3:**
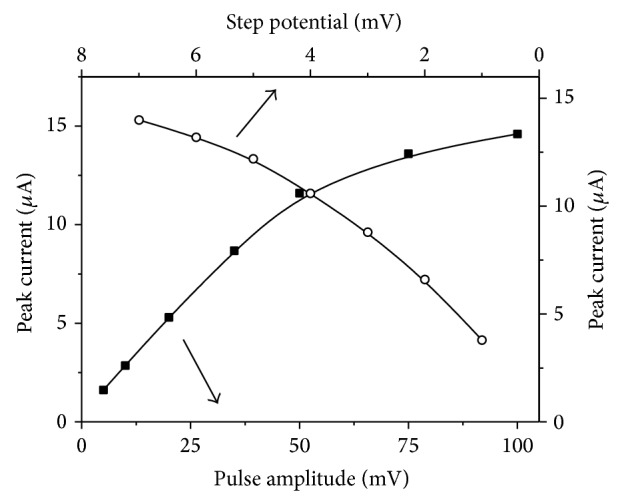
Dependence of the peak current on pulse amplitude in the range of 10 to 100 mV and step potential in the range of 1 to 7 mV for 10 *μ*M codeine in 0.1 M phosphate buffer (pH 3.0) volume of Nafion/MWCNTs 10 *μ*L. Other instrumental parameters: *t*_*w*_, *t*_*s*_ = 20 ms, preconcentration potential 300 mV, preconcentration time 20 s, and stirring rate 500 rpm.

The best results were obtained for the amplitude of 50 mV (the peak current was ~12 *μ*A for 10 *μ*M codeine, Figure 3). Higher pulse amplitude (>50 mV) caused the growth of the background current practically without increase of the peak current. For further work, the pulse amplitude of 50 mV was applied.

Changes of the step potential cause influence on peak current. For a step potential equal to 1 mV the peak current was 3.8 *μ*A, and for a step potential of 7 mV the peak current was 14 *μ*A (Figure 3). The step potential of 5 mV was applied in further work (good relation codeine signal to background current).

**Figure 4:**
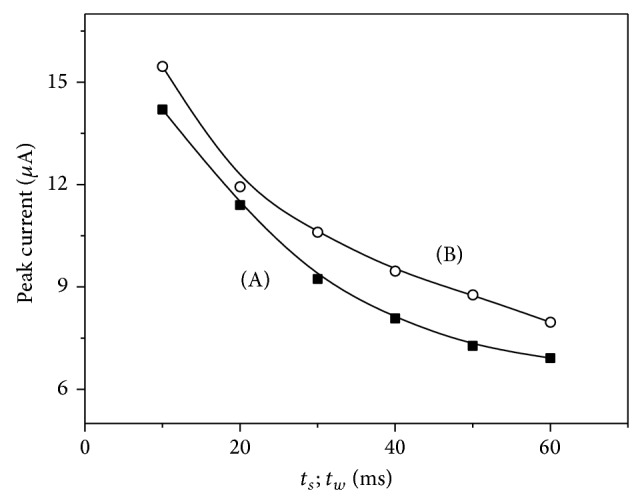
Dependence of the peak current on sampling (A) and waiting time (B) in the range of 10 to 60 ms for 10 *μ*M codeine in 0.1 M phosphate buffer (pH 3.0) volume of Nafion/MWCNTs 10 *μ*L. Other instrumental parameters: Δ*E* = 50 mV, *E*_*s*_ = 5 mV, preconcentration potential 300 mV, preconcentration time 20 s, and stirring rate 500 rpm.

The waiting time and sampling time were changed in the range from 10 to 60 ms (Figure 4). The best result was obtained for waiting time and sampling time of 20 ms (best relation signal to background current), and this was the value chosen for further work.

### 3.3. Influence of the Volume of Nafion/MWCNTs

**Figure 5:**
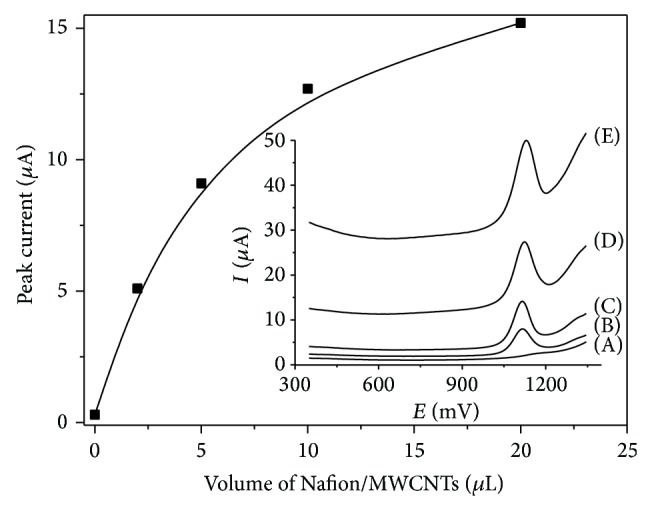
Dependence of the peak current on volume of Nafion/MWCNTs on GC electrode in the range of 0 to 20 *μ*L for 10 *μ*M codeine in 0.1 M phosphate buffer (pH 3.0) and obtained voltammograms for (A) 0; (B) 2; (C) 5; (D) 10; (E) 20 *μ*L Nafion/MWCNTs. Instrumental parameters: Δ*E* = 50 mV, *E*_*s*_ = 5 mV, and *t*_*w*_, *t*_*s*_ = 20 ms. Preconcentration potential 300 mV, preconcentration time 20 s, and stirring rate 500 rpm.

The mixture of Nafion/MWCNTs coating the GC electrode is necessary to obtain a high sensitive determination of codeine. The codeine peak current depends on the volume of Nafion/MWCNTs (Figure 5).

For bare GC electrode the codeine peak current was 0.3 *μ*A. Presence and increase of the amount of Nafion/MWCNTs on the GC electrode are accompanied by an increase of the codeine peak. The optimal volume of Nafion/MWCNTs was 10 *μ*L (with the peak current reaching values about 12 *μ*A). Higher volumes of Nafion/MWCNTs cause increase in a background current and worse repeatability of the codeine signal. The presence of Nafion/MWCNTs also had an influence on the peak potential. For bare GC electrode the DPV codeine peak potential was 1180 mV and for modified electrode with Nafion/MWCNTs the codeine peak potential was 1125 mV. The negative shift of the peak potential suggests catalytic effect caused by Nafion/MWCNTs. For further work, the volume of 10 *μ*L was used.

### 3.4. Influence of Preconcentration Potential and Time on Codeine Peak

The influences of preconcentration potential and time are usually important factors on the sensitivity and detection limit of the stripping methods. Preconcentration potential for codeine determination in 0.1 M phosphate buffer (pH 3.0) was investigated in the range from −200 to 900 mV. The experiment showed that the preconcentration potential has no influence on the codeine peak current. In the whole work arbitrary the 300 mV preconcentration potential was used.

**Figure 6:**
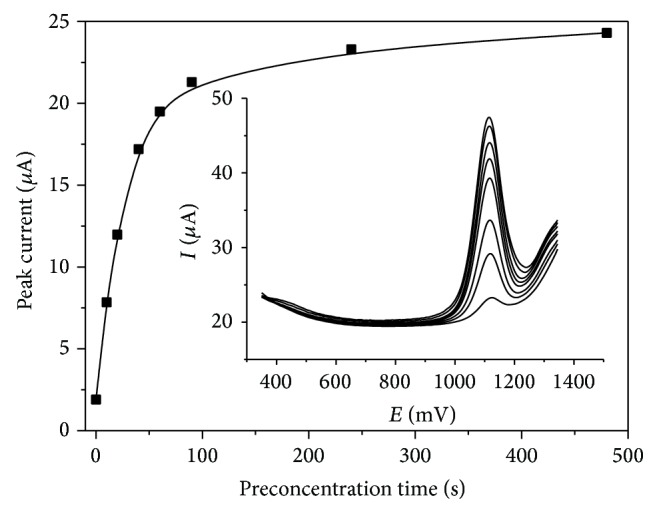
Dependence of the peak current on preconcentration time in the range from 0 to 480 s for 10 *μ*M codeine in 0.1 M phosphate buffer (pH 3.0) and volume of Nafion/MWCNTs 10 *μ*L and obtained voltammograms. All other conditions are as in Figure 5.

The changes in magnitude of the codeine current versus preconcentration time are presented in Figure 6.

The peak current increased with the increase of the preconcentration time from 1.9 *μ*A (*t*_acc_ = 0 s) to 24.3 *μ*A (*t*_acc_ = 480 s). For a preconcentration time higher than 120 s, practically no increase of the codeine peak current was observed. The codeine peak potential is independent of the preconcentration potential.

### 3.5. Influence of Electrolyte Composition and pH on Codeine Peak

**Figure 7:**
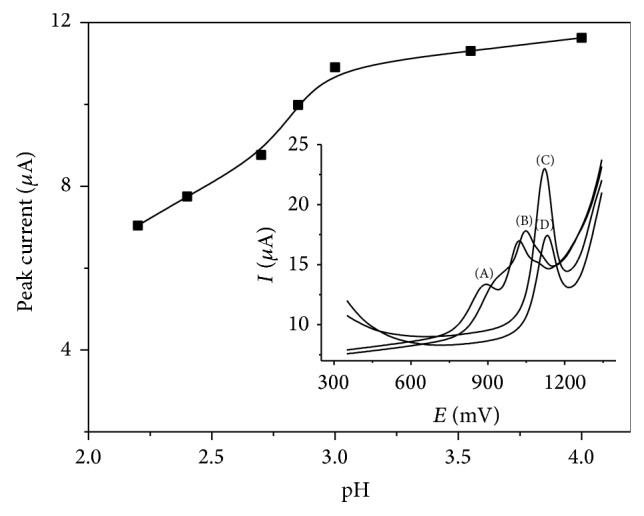
Dependence of the peak current on pH (2.2; 2.4; 2.7; 2.8; 3; 3.6; 4.0) for 10 *μ*M codeine in 0.1 M phosphate buffer and volume of Nafion/MWCNTs 10 *μ*L and obtained voltammograms for (A) 8.0; (B) 7.5; (C) 3.0; (D) 2.2 pH. All other conditions are as in Figure 5.

The electrochemical oxidation of codeine has been studied in 0.1 M KCl (pH 6.8), KNO_3_ (pH 6.9), acetate buffer (pH 3.8), borate buffer (pH 9.1), and phosphate buffers (pH in the range of 2.2 to 8.0). The best results were obtained in phosphates. Determination of codeine on GC electrode modified with Nafion/MWCNTs in phosphates requires an acidic condition in order to obtain a single peak. The shape of codeine peak depends strongly on the pH. For the pH in the range of 8.0 to 7.5 triple codeine peak was observed and for the pH in the range of 7.0 to 4.8 double codeine peak was observed. Single, analytical, suitable, and well-shaped codeine signal was observed for the pH lower than 4.8 and this suggests that the anodic oxidation of codeine follows a complex mechanism that is pH dependent. The optimal pH for the quantity determination of small amounts of codeine was in the range from 4.0 to 3.0 (peak current reaching value about 12 *μ*A for 10 *μ*M codeine). More acidic conditions than pH 3.0 caused a decrease in the peak current; for example, for the pH of 2.2 the peak current was 7 *μ*A (Figure 7). For further measurements, the pH of 3.0 was applied.

### 3.6. Interference

The examined ions, such as Ca(II), Mg(II) in a 1000-fold excess, Zn(II), Mn(II) in a 100-fold excess, and Pb(II), Cd(II), and Cu(II) in a 2-fold excess, did not interfere. Organic compounds such as caffeine, citric acid, and ascorbic acid in a 20-fold excess and glucose 25 mg·L^−1^ did not interfere.

The surface-active compounds are usually a source of strong interference in voltammetric methods. A nonionic surface-active compound (Triton X-100) was investigated in this respect. For 0.25 mg·L^−1^ of Triton X-100 concentration, no suppression of the signal was observed. Higher concentration of Triton X-100 caused suppression of the signal, for example, for 2.5 mg·L^−1^ of Triton X-100 by 50% and for 6 mg·L^−1^ of Triton X-100 by 70%.

### 3.7. Analytical Performance

**Figure 8:**
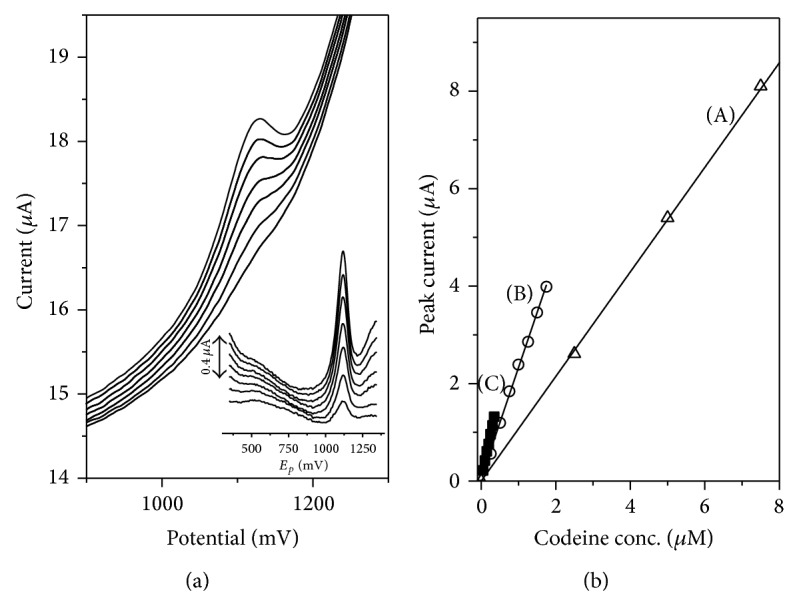
(a) The DP SV codeine calibration voltammograms for 0.05, 0.1, 0.15, 0.2, 0.25, 0.3, and 0.35 *μ*M codeine obtained for preconcentration time 120 s in 0.1 M phosphate buffer (pH of base electrolyte 3.0) and volume of Nafion/MWCNTs 10 *μ*L and (b) codeine calibration curves obtained for preconcentration times of (A) 15, (B) 60, and (C) 120 s. All other conditions are as in Figure 5.

The DP SV voltammograms of codeine for the 0.05–0.35 *μ*M concentration range and preconcentration time of 120 s are presented in Figure 8.

The detection limit obtained for short preconcentration time (15 s) was 130 nM with the linearity up to 15 *μ*M (slope of the regression line was 1.02 ± 0.03 (*μ*A·*μ*M^−1^), intercept 0.21 ± 0.23 *μ*A, and correlation coefficient 0.998). A longer preconcentration time results in a lower detection limit (e.g., when the preconcentration time of 60 s was used during the measurement the detection limit was 22 nM, and for the preconcentration time of 120 s the detection limit was 14 nM).

The slopes for regression lines were (*μ*A·*μ*M^−1^) 2.27 ± 0.02 and 3.67 ± 0.05, intercepts (*μ*A) 0.21 ± 0.22 and 0.04 ± 0.03, and the correlation coefficients 0.996 and 0.997 for preconcentration times of 60 and 120 s, respectively. The linearity was up to 1.75 *μ*M (*t*_acc_ = 60 s) and 0.5 *μ*M (*t*_acc_ = 120 s).

To validate the method the urine, human plasma, and drugs were investigated.

The samples, spiked with codeine, were analyzed according to the described procedure using the GC electrode modified with Nafion/MWCNTs. Determinations of codeine were performed using the standard addition method (three additions of the standard solution). Results from codeine determination are presented in Table 1. The recovery of codeine ranged from 92 to 105%. The analytical usefulness of the presented method for the determination of codeine in the samples was confirmed.

## 4. Conclusions

The presented* DPV* method for the electrochemical determination of codeine using a GC electrode modified with Nafion/MWCNTs allows determining codeine at trace level, in concentrations as low as 14 nM (4.5 *μ*g·L^−1^), calculated according to [25] for a preconcentration time of 120 s. The reproducibility of the method is very good; that is, when measured as RSD it is 3.7%. Acceptable recovery (92–105%) shows that the method can be used for the determination of codeine in drugs and human body fluids.

The preparation of GC electrode modified with Nafion/MWCNTs is very simple, short, and economically acceptable. The obtained results confirm that method may be used in out-of-laboratory systems.

Voltammetric responses of Nafion/MWCNTs in terms of linear range and detection limits were compared to the other electrodes reported in the literature (Table 2).

## Figures and Tables

**Table 1:** Results of codeine determination in various samples.

Codeine added	Codeine found $\overset{-}{x}\pm s$ (recovery %)			
	Urine (mg/L)	Human plasma (mg/L)	Syrup^1^ (mg/10 mL)	Tablet^2^ (mg/tablet)
0	0	0	15.5 ± 0.7	15.3 ± 0.4

0.15 mg/L	0.144 ± 0.009 (96)	0.138 ± 0.012 (92)	—	—

0.6 mg/L	0.632 ± 0.027 (105)	0.57 ± 0.03 (95)	—	—

15 mg	—	—	31.4 ± 1.1 (103)	30.9 ± 0.9 (102)

^1^Product declared 15 mg/10 mL.

^2^Product declared 15 mg/tablet.

**Table 2:** Voltammetric detection of codeine reported at various electrodes.

Electrode	Linear range	Detection limit	Reference
CME	0–32 *µ*M	10 nM	[17]
CMSPE	2.5–45 *µ*M	20 nM	[18]
PB/Pd/Al	2–30 *µ*M	0.8 *µ*M	[19]
Pd/Al	0.1–3 mM	5 *µ*M	[20]
Boron-doped diamond	0.1–60 *µ*M	80 nM	[22]
SWCNTs/CCE	0.2–230 *µ*M	0.11 *µ*M	[23]
Nafion/MWCNTs (*t*_acc_ = 120 s)	0–0.5 *µ*M	14 nM	This work
